# Foliar Nutritional Quality Explains Patchy Browsing Damage Caused by an Invasive Mammal

**DOI:** 10.1371/journal.pone.0155216

**Published:** 2016-05-12

**Authors:** Hannah R. Windley, Mandy C. Barron, E. Penelope Holland, Danswell Starrs, Wendy A. Ruscoe, William J. Foley

**Affiliations:** 1 Research School of Biology, The Australian National University, Acton, ACT 2601, Australia; 2 Landcare Research, PO Box 69040, Lincoln, 7640, New Zealand; College of Agricultural Sciences, UNITED STATES

## Abstract

Introduced herbivores frequently inflict significant, yet patchy damage on native ecosystems through selective browsing. However, there are few instances where the underlying cause of this patchy damage has been revealed. We aimed to determine if the nutritional quality of foliage could predict the browsing preferences of an invasive mammalian herbivore, the common brushtail possum (*Trichosurus vulpecula*), in a temperate forest in New Zealand. We quantified the spatial and temporal variation in four key aspects of the foliar chemistry (total nitrogen, available nitrogen, *in vitro* dry matter digestibility and tannin effect) of 275 trees representing five native tree species. Simultaneously, we assessed the severity of browsing damage caused by possums on those trees in order to relate selective browsing to foliar nutritional quality. We found significant spatial and temporal variation in nutritional quality among individuals of each tree species examined, as well as among tree species. There was a positive relationship between the available nitrogen concentration of foliage (a measure of *in vitro* digestible protein) and the severity of damage caused by browsing by possums. This study highlights the importance of nutritional quality, specifically, the foliar available nitrogen concentration of individual trees, in predicting the impact of an invasive mammal. Revealing the underlying cause of patchy browsing by an invasive mammal provides new insights for conservation of native forests and targeted control of invasive herbivores in forest ecosystems.

## Introduction

Invasive herbivores can have significant deleterious impacts on ecosystems. However, these impacts can vary greatly across a landscape. For instance, invasive herbivores often inflict patchy damage on plant communities, resulting in the mortality of some trees while others remain unaffected [1]. A wide range of intrinsic, extrinsic and community-level factors shape both the success of the invader, and the resilience of the invaded ecosystem [2]. These factors include the relative vulnerability of plants to novel herbivores [3], the release of the herbivore from top-down controls such as natural predators or parasites [4] and the availability of resources such as food and den sites [2]. However, these factors do not always explain the observed spatiotemporal variation in damage inflicted by invasive herbivores. Previous studies examining natural plant-herbivore interactions have revealed that underlying nutritional quality across numerous spatial scales (from individual trees to landscapes) can explain the browsing decisions, and more generally, the population dynamics of herbivores (e.g., [5–12]). While the browsing decisions of herbivores in their native range can be explained by nutritional quality, the extent to which this influences herbivore browsing decisions when they are invasive is unknown.

The common brushtail possum (*Trichosurus vulpecula*) in New Zealand presents an excellent study system to test if foliar nutritional quality can explain the browsing decisions of an invasive, herbivorous mammal. The common brushtail possum is a ~2–6 kg marsupial that was introduced to New Zealand (NZ) from Australia in the 1850s [13]. Since introduction, they have caused extensive heterogeneous damage to native NZ forests [14–16]. Primarily folivorous, possums are a major cause of defoliation and contribute to mortality in many native NZ plant species, particularly dominant tree species such as rata (*Metrosideros spp*.), kamahi (*Weinmannia racemosa*) and mahoe (*Melicytus ramiflorus*) [15, 17–21]. However, the extent to which browsing by possums contributes directly and indirectly to tree mortality in NZ has been widely debated [15, 22, 23]. Nonetheless, the replacement of locally palatable dominant tree species with less palatable species may have wider ecosystem consequences [15, 24, 25]. Understanding the underlying drivers of browsing decisions by possums is important for both prioritizing possum management actions, and supporting the conservation of native forests in New Zealand. It has been hypothesized that nutritional quality may help explain the browsing decisions of brushtail possums in New Zealand [26], but this has not been tested. Previous studies have used tree size as a proxy for tree preference by possums in NZ [23, 26], but evidence from Australia suggests that nutritional quality of tree foliage may be a better predictor (e.g., [8, 10, 11]).

Difficulties in relating foliar nutritional quality to browsing preferences are due to the many dietary factors that can influence feeding decisions. One approach for measuring the nutritional quality of leaves that accounts for protein, tannins and fibre is the *in vitro* available nitrogen assay, termed ‘AvailN’ [27]. The *in vitro* digestion and simultaneous measurement of the effect of tannins using polyethylene glycol 4000 (PEG), allows the estimation of the effects of tannins on nitrogen availability [11, 28] as well as the *in vitro* dry matter digestibility of the sample. AvailN provides the most functional and integrative measure of nutritional quality for mammalian folivores currently available. DeGabriel et al. [29] used this assay to show that the effect of tannins on the availability of protein was ultimately determining the nutritional value of foliage to a population of wild common brushtail possums (*Trichosurus vulpecula*) in northern Australia. More importantly, DeGabriel et al. [29] demonstrated significant spatial variation in AvailN at landscape scales. Information regarding the variation in nutritional quality at a landscape scale is required to assess if nutritional quality can be used to explain the distribution and abundance of invasive herbivores.

This study aimed to determine if there is a relationship between browsing damage caused by possums and the nutritional quality of dominant tree species in the Tararua Mountain Range, NZ (hereafter ‘the Tararuas’). We tested the hypothesis that AvailN concentration in leaves would vary both spatially and temporally among individuals of the same tree species, and among tree species, and that this variation in nutritional quality influences the browsing decisions of possums. We hypothesised that if nutritional factors are driving the patchy damage caused by browsing by possums in the Tararuas, trees and tree species that have foliage with high AvailN will exhibit greater severity of possum damage than those with low AvailN. Finally, we aimed to determine the effect of possum abundance on their browsing decisions; hypothesizing that in sites where possum abundance is high, there is greater demand for high quality foliage and thus greater damage to trees with high foliar AvailN.

## Materials and Methods

### Collection site and sampling design

The Tararua Mountain Range (40.77° S, 175.38° E) runs in a northeast-southwest direction for 80 km from near Palmerston North to the Hutt Valley in NZ. The height of the Tararua summits range between 1300 and 1500 m. Sampling was conducted along two 5 km transects (referred to as Line 1 and Line 2 hereafter). The two lines run roughly parallel, either side of the range, and are approximately 15 km apart (S1 Fig), with 25 sampling plots located every 250 m along each line. Routine possum control in the Tararua Mountain Range was conducted by the New Zealand Department of Conservation, commencing in Spring 2010, as part of nation-wide pest control operations. Each line traversed the control area boundary with plots 1–13 (Line 1) and 26–38 (Line 2) contained within the boundary (S1 Fig). In order to assess the effects of the pest control, relative possum abundance was measured using lured wax-blocks (WaxTags^®^ Pest Control Research LP) located along ten 200 m long transects (20 WaxTags per transect) per line (*sensu* NPCA protocol [30]). The proportion of WaxTags per transect that were bitten by possums (bite mark index) was recorded at the same time that assessments were made of browsing by possums (described below).

We sampled trees at each plot on each line, with the number of trees sampled reflecting their relative abundance at each plot (S1 Table). Based on its high local relative abundance, and previous research [26, 31, 32], *Weinmannia racemosa* (kamahi) was the focal tree species for sampling in this study and at least one kamahi tree was sampled from each plot. Permission to collect foliage was obtained from the Department of Conservation, New Zealand (National Permit Number WE/2794/RES).

### Foliage sampling

Sampling occurred over 10 days each season. Samples were collected in November (Spring) 2010, February (Summer) 2011, May (Autumn) 2011 and November (Spring) 2011. The same 275 trees were sampled each season (S1 Table). We collected leaf samples from five tree species on Line 2. The tree species were kamahi, *Myrsine salicina* (toro), *Dacrydium cupressinum* (rimu), *Elaeocarpus dentatus* (hinau) and *Melicytus ramiflorus* (mahoe) but only kamahi, toro and rimu were present at Line 1. Tree basal area (TBA; m^2^) was calculated from the diameter at breast height of each tree, which was measured in November 2010. We removed branches from the canopy with a shotgun, plucked approximately 50 g (wet weight; w/w) of mature leaf from the fallen branch and the sample was placed in a plastic zip-lock bag. The samples were immediately frozen on a bed of solid CO_2_ pellets in the field and remained frozen during transport and storage before being freeze dried. We ground the dried samples to pass a 1 mm sieve in a Tecator Cyclotec Mill (Foss, Hillerød, Denmark) and stored samples in sealed containers at 5°C in the dark before collection of near infrared reflectance spectra (described below).

### Assessment of browsing by possums

The extent of herbivory by possums on each sampled tree was assessed using a method based on the Foliar Browse Index (FBI; [33]). This involved a ground-based visual assessment of the tree canopy for evidence of browsing. Each tree was allocated a category indicating the extent of browsing by possums as: No browse = 0%, Light browse = 0–5%, Moderate = 6–25%, Heavy = 26–100%. These percentage ranges differ from those defined by Payton et al. [33], in that we combined some categories, reducing the number of browse categories from six to four.

### Analysis of nutritional parameters in foliage

Total nitrogen (total N) was measured using a Dumas procedure on a LECO TruSpec^®^ CN analyser (LECO, St Joseph, MI, USA). We used the available nitrogen assay described by DeGabriel et al. [27] to measure the other traits: available nitrogen, (AvailN); available nitrogen adjusted for tannins via incubation with polyethylene glycol (PEG), (AvailNP); and *in vitro* dry matter digestibility, (DMD). This method requires a ground sample to pass through a series of incubations that broadly mimic the digestion of a hindgut-fermenting herbivore. Incubation with PEG releases protein from tannin–protein complexes and enables the measurement of the effect of tannins on protein availability. Hereafter, ‘tannin effect’ refers to the amount of nitrogen in a sample that could be bound by tannins, and was calculated by subtracting AvailN from AvailNP. All traits are presented as percentage of dry matter.

### Near infrared reflectance spectroscopy (NIRS)

We developed calibrations using NIRS to predict the concentrations of the four nutritional parameters; total N, AvailN, AvailNP and DMD. This calibration procedure is covered in detail in Windley and Foley [34]. Briefly, we scanned the samples (n = 1399) with a Bruker MPA Fourier Transform NIR spectrophotometer (Bruker Optik GmbH, Ettlingen, Germany) and imported the spectra to the WinISI III software package (Infrasoft International, Port Matilda, PA, USA). We identified samples to make up a calibration set (*n* = 650) and a validation set (*n* = 50) using the WinISI package as described by Shenk and Westerhaus [35]. We analysed the nutritional parameters in the foliage as described above in both sets of samples and a GLOBAL calibration procedure was employed to perform the predictions of nutritional parameters for the remaining samples (*n* = 699).

### Statistical analysis

To develop an understanding of the underlying causes of variation in possum browsing damage in the Tararuas, we examined sources of variation in nutritional quality of foliage with respect to the effects of line, season, species and TBA on each nutritional parameter, separately. Variation in foliar nutritional quality has been extensively explored in Windley and Foley [34]. Firstly, to explore species-level differences in nutritional quality, each response variable (N, AvailN, tannin effect and DMD) was modelled using mixed-effects linear regression with tree species as the fixed variable. Tree ID nested within plot were included as random factors to control for plot-level variance, and the repeated nature of sampling from the same tree across the four seasons. Significant differences between species were identified using post-hoc Tukey HSD pairwise comparisons. To identify the subset of significant predictors of variation in each nutritional parameter, forward model selection was employed using log-ratio tests, in which fixed variables (species, line, season, TBA) were added in turn, and the variable that resulted in the greatest change in the log-ratio statistic was retained, and the procedure repeated with remaining non-fitted variables. The optimal model was determined when no additional variables or all possible interaction terms explained a significant proportion of the variance (determined from log-ratio tests). All analyses were performed in R (version 3.01, [36]) using the package ‘lme4’ (version 1.0–4, [37]).

After examining the sources of variation in foliar nutritional quality, we examined the browsing behaviour of possums in relation to nutritional quality using cumulative link mixed effects models (CLMM). CLMM can be applied where the response variable consists of multiple ordinal, categorical values (in this case, 4 browse categories, in which no browse < light browse < moderate browse < heavy browse). The R package ‘ordinal’ (version 2013.9–30, [38]) allows for the construction of models with many similarities to GLM models, including fixed and random factors. The tree species rimu was dropped from the analysis as no browsing damage by possums was observed on this species at our study site. Likewise, samples from Line 1 were excluded from analysis due to substantial differences in both damage from browsing by possums and relative possum abundance between the two Lines (e.g., 99% of trees sampled at Line 1 were not browsed by possums). The set of explanatory variables available were season, tree species, control, TBA and each of the nutritional parameters. Bite mark index data indicated that baiting and trapping in the control plots reduced possum densities over the duration of the study. However, saturation of the wax tags occurred over the duration of the study at uncontrolled plots at Line 2. Saturation of the wax tags acts as a form of data censoring, as variation in possum abundance above that required to achieve saturation, is unobserved. As such, control was treated as a two level factor (control/no control), instead of using a quantitative possum density index, *sensu* Duncan et al. [26]. Forward model selection was conducted using log-ratio tests as outlined above. We tested each nutritional parameter in turn, rather than including all within the same model, as AvailN and total N were strongly, positively correlated.

Kamahi was selected for further analysis, because of its relative abundance and frequently browsed status at the study site, and because it is considered to be a fundamental species in possum diet [26, 32]. Restricting analysis to just kamahi, CLMM was employed with possum browse category as the response variable, and potential explanatory variables (season, TBA, control and AvailN) were added in turn and log ratio tests used to identify the best model. As above, only samples from Line 2 were used in this analysis.

## Results

### Variation in foliar nutritional quality in the Tararua Mountain Range

**Fig 1:**
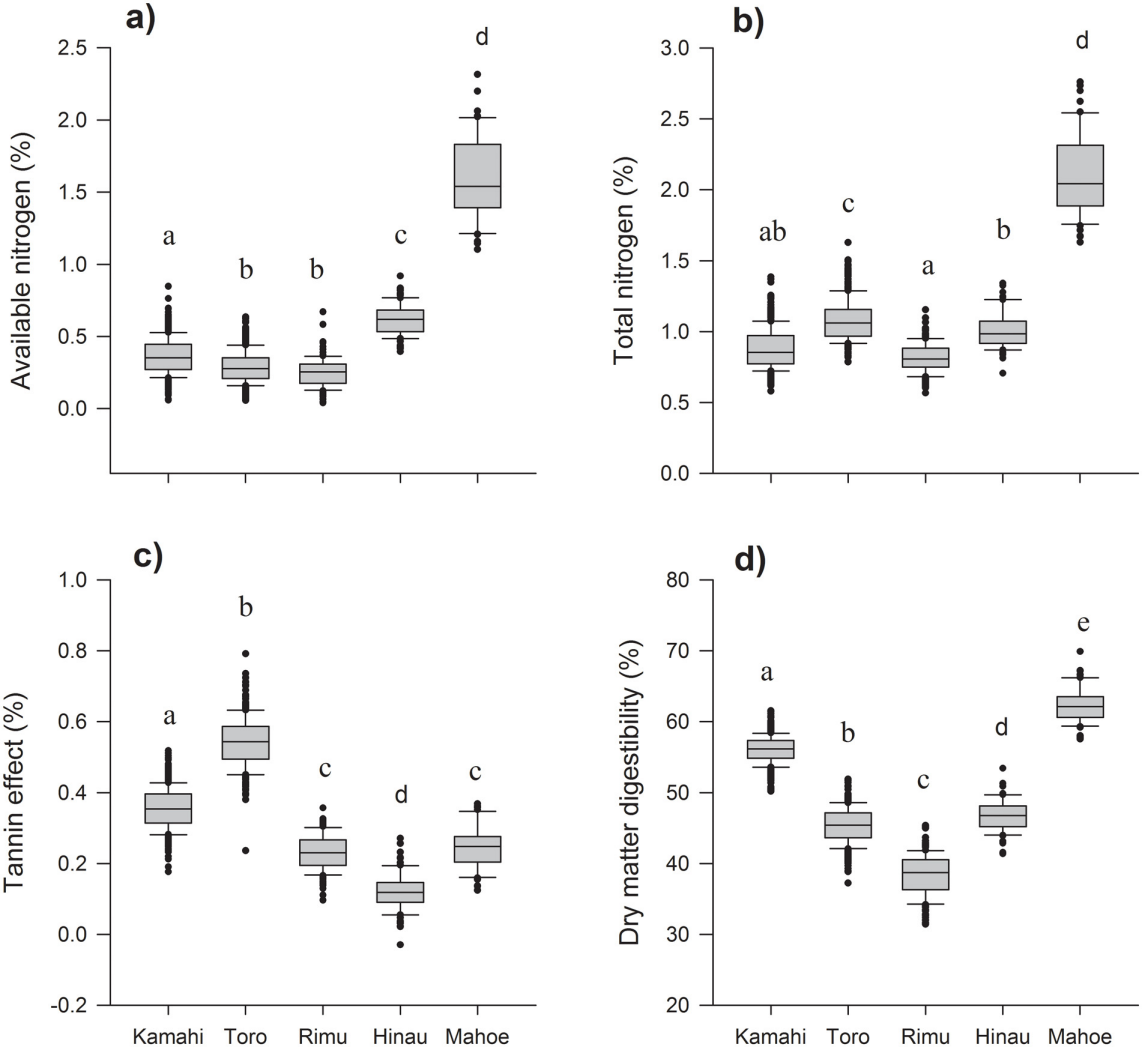
Nutritional characteristics of foliage. Box and whisker plots of measured a) available nitrogen, b) total nitrogen (N), c) tannin effect and d) dry matter digestibility of foliage from five tree species in the Tararua Mountain Range, New Zealand). Letters denote significant differences between species (Tukey HSD). (*n* = 1111).

There were significant differences between tree species for average AvailN concentration (P<0.001), total N concentration (P<0.001), DMD (P<0.001) and tannin effect (P<0.001) (Fig 1, S2 Table). Tukey post-hoc testing revealed that mahoe foliage had higher average AvailN concentrations than all other species, followed by hinau, then kamahi. Rimu and toro foliage had the lowest AvailN concentrations (Fig 1a). Total N concentrations were highest in mahoe, followed by toro. Total N concentrations in hinau and kamahi and rimu and kamahi foliage were not significantly different (Fig 1b). The tannin effect was highest in toro, followed by kamahi, then mahoe and rimu, with hinau having the lowest tannin effect (Fig 1c). DMD was highest for mahoe, followed by kamahi, hinau and toro. Rimu had the lowest DMD (Fig 1d).

The nutritional quality of foliage (AvailN, total N, tannin effect and DMD) in the Tararuas varied through space and time and among species (Fig 1), with numerous interactions between line, season and species (S3 Table, and also see Windley and Foley [34]). Tree basal area (TBA) was a significant predictor of foliar DMD, but not for any of the other nutritional factors (S3 Table). Foliage at Line 1 was significantly lower in AvailN and total N than that at Line 2 for kamahi, toro and rimu, with the average AvailN concentration of kamahi at Line 1 and Line 2 being 0.28% ± 0.08 SE and 0.40% ± 0.09 SE, respectively (S2 Fig). Generally, the AvailN concentration and DMD concentration of foliage was highest in Spring and lowest in Autumn, while total N concentration and tannin effect was highest in Autumn and lowest in Spring (S2 Fig).

### Browsing by possums and foliar nutrition in the Tararua Mountain Range

We found a significant positive relationship between browsing by possums and foliar AvailN and total N in models containing all tree species at Line 2 (Tables 1 and 2). Season, species, possum control and their interactions also had a significant effect on browsing by possums (Table 1). There was no significant relationship between browsing by possums and TBA and adding this measure to the model did not produce a significant improvement in model fit (Table 1). Total N and AvailN are highly correlated, so although both had a significant effect on browsing by possums (when modeled separately; Table 1), we focused our analysis on AvailN as it is a more integrative measure of nutritional quality. Kamahi was the tree species with the strongest relationship between possum browsing and foliar nutritional quality, and there were significant interactions between season and possum control and season and AvailN on browsing by possums (Tables 1 and 2).

**Table 1:** Summary of model selection.

a)					
**AvailN Model**	**Log likelihood**	**k**	**LR.df**	**LR stat**	**P**
Intercept (Null model)	-533.45	5			
Season	-511.96	8	3	42.978	<0.001
Season + Species	-497.07	11	3	29.779	<0.001
Season + Species + Control	-492.86	12	1	8.4177	0.004
Season + Species + Control + AvailN	-489.77	13	1	6.1743	0.013
Season + Species + Control + AvailN + AvailN * Season	-483.01	16	3	13.582	0.004
Season + Species + Control + AvailN + AvailN * Season + Season * Control	-475.64	19	3	14.734	0.002
Season + Species + Control + AvailN + AvailN * Season + Season * Control + Species * Control	**-470.55**	**22**	**3**	**10.18**	**0.017**
b)					
**Total N Model**	**Log likelihood**	**k**	**LR.df**	**LR stat**	**P**
Intercept (Null model)	-533.45	5			
Season	-511.96	8	3	42.98	<0.001
Season + Species	-497.07	11	3	29.78	<0.001
Season+Species+N	-491.89	12	1	10.35	0.001
Season+Species+N+Control	-487.71	13	1	8.38	0.003
Season+Species+N+Control+Season*Species	-475.51	22	9	24.39	0.004
Season+Species+N+Control+Season*Species+Season*Control	-468.85	25	3	13.33	0.004
Season+Species+N+Control+Season*Species+Season*Control+Species*Control	-463.05	28	3	11.58	0.009
Season+Species+N+Control+Season*Species+Season*Control+Species*Control+N*Species	**-457.58**	**31**	**3**	**10.94**	**0.012**
c)					
**Tannin Model**	**Log likelihood**	**k**	**LR.df**	**LR stat**	**P**
Intercept (Null model)	-533.45	5			
Season	-511.96	8	3	42.98	<0.001
Season + Species	-497.07	11	3	29.78	<0.001
Season+Species+Control	-492.86	12	1	8.42	0.004
Season+Species+Control+Season*Species	-481.47	21	9	22.78	0.007
Season+Species+Control+Season*Species+Season*Control	-474.84	24	3	13.25	0.004
Season+Species+Control+Season*Species+Season*Control+Species*Control	**-469.58**	**27**	**3**	**10.52**	**0.015**
d)					
**DMD Model**	**Log likelihood**	**k**	**LR.df**	**LR stat**	**P**
Intercept (Null model)	-533.45	5			
Season	-511.96	8	3	42.98	<0.001
Season + Species	-497.07	11	3	29.78	<0.001
Season+Species+Control	-492.86	12	1	8.42	0.004
Season+Species+Control+Season*Species	-481.47	21	9	22.78	0.007
Season+Species+Control+Season*Species+Season*Control	-474.84	24	3	13.25	0.004
Season+Species+Control+Season*Species+Season*Control+Species*Control	**-469.58**	**27**	**3**	**10.52**	**0.015**

Summary of forward model selection of cumulative link mixed effects model for possum browsing as a function of environmental and nutritional factors using log ratio statistics. Explanatory variables were added to the null model of Browse ~ 1 in turn, and the variable with the most explanatory power (determined by log ratio statistic) retained, and the procedure repeated with remaining, unfitted variables. The random factors (Plot, and tree, nested within Plot) were present in all models. The term k refers to the number of coefficients in the model; LR. df is the log ratio statistic degrees of freedom. LR stat. is the log ratio statistic; P is the P value associated with the log ratio statistic where a P value <0.05 indicates a significant difference between the model and the preceding model (i.e., a significant improvement in the model). The final model is highlighted in bold, defined by the condition that the addition of no other variables or interaction terms produced a significant improvement in model fit.

**Table 2:** Summary of model coefficients.

**Coefficient**	**Estimate**	**SE**	**Wald Z**	**P**
Summer	-0.445	0.595	-0.749	0.454
Autumn	-0.264	0.596	-0.413	0.679
Spring 2011	0.804	0.706	1.138	0.255
**kamahi**	**5.112**	**0.909**	**5.624**	**<0.001**
mahoe	-0.343	1.733	-0.198	0.843
**toro**	**3.288**	**1.071**	**3.071**	**0.002**
ControlY	-0.305	1.226	-0.249	0.804
**AvailN**	**4.380**	**1.356**	**3.231**	**0.001**
Summer *AvailN	-0.116	0.712	-0.163	0.871
Autumn *AvailN	-0.812	0.730	-1.119	0.263
Spring 2011*AvailN	**-3.363**	**1.029**	**-3.267**	**0.001**
Summer*Control	-0.663	0.558	-1.188	0.235
Autumn *Control	-0.633	0.557	-0.114	0.910
Spring 2011*Control	**-2.145**	**0.642**	**-3.341**	**<0.001**
kamahi *Control	-1.714	1.275	-1.344	0.179
mahoe *Control	-0.496	1.665	-0.298	0.766
toro *Control	1.473	1.428	1.032	0.302
**Random Factor**	**Variance**			
Plot	0.383			
Plot/Tree	2.96			

Summary of model coefficients from cumulative link mixed effects modelling exploring the fixed effects of season, species, possum control, AvailN and significant interactions, on severity of possum browse. Note: Spring 2010, hinau and non-control zone are the reference levels for their respective factor. Hence, significant differences (marked in bold text) refer to significant differences from reference levels. Estimate is the coefficient estimate; SE is the standard error of the estimate; Wald Z is the Wald Z statistic; P is the P value associated with the Wald Z statistic. Significant differences from the reference condition are denoted by bold case.

**Fig 2:**
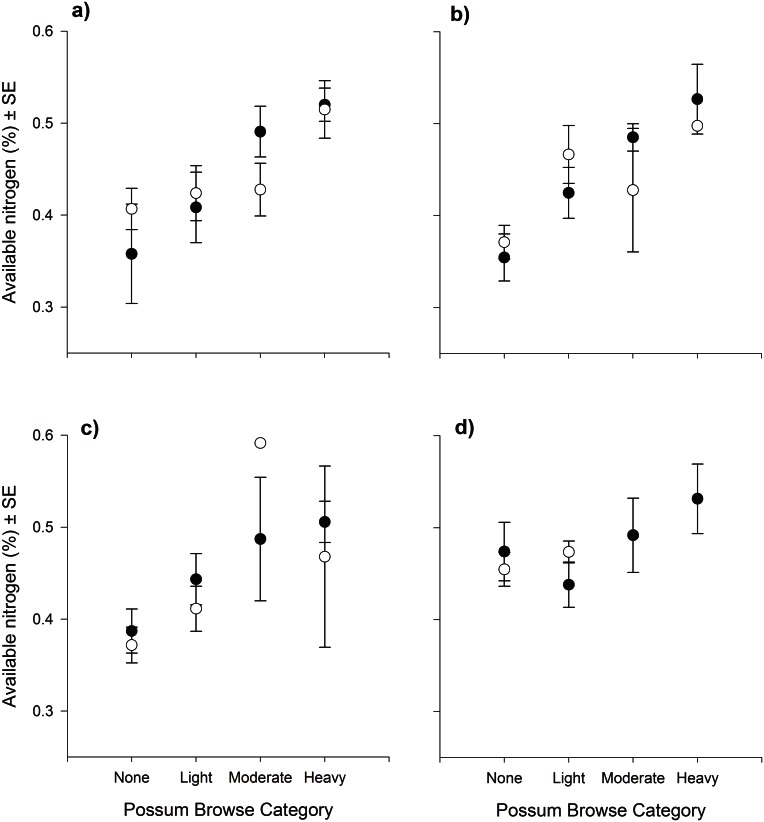
Relationship between kamahi foliar AvailN and possum browse category. Relationship between the available nitrogen concentration (mean ± SE) of kamahi foliage and possum browse category at Line 2 for trees in possum control zone (open circles) and non-control zone (closed circles) during a) Spring 2010; b) Summer 2011; c) Autumn 2011; d) Spring 2011. Note the single data point in panel c for the moderate browse category.

There was very little browsing damage by possums at Line 1 possibly related to the significantly lower AvailN at that line (see above). Season, possum control, total N and AvailN all had a significant effect on browsing by possums of kamahi at Line 2, while DMD and tannin effect did not have a significant effect on browsing by possums (Table 3). Notably in Spring 2011, browsing damage by possums on kamahi was significantly less severe compared to the preceding seasons (Fig 2, Table 4). The average AvailN of the target kamahi trees was higher in Spring 2011 than in previous seasons and corresponded with a much narrower range of AvailN values than in previous seasons (Fig 2). The relationship between AvailN and possum browsing severity was weaker (though not significantly) at sites where possum control occurred (i.e., where possum abundance was reduced) compared with sites where there was no possum control (i.e., where possum abundance was high) (Fig 2, Table 4).

**Table 3:** Summary of model selection, possum browsing on kamahi at Line 2.

a)					
**AvailN Model**	**Log Likelihood**	**k**	**LR.df**	**LR stat**	**P**
Intercept (Null model)	-285.15	5			
Season	-272.98	8	3	24.34	**<0.001**
Season +AvailN	-266.07	9	1	13.84	**<0.001**
**Season + AvailN + Control**	**-259.6**	**10**	**1**	**12.94**	**<0.001**
b)					
**Total N Model**	**Log likelihood**	**k**	**LR.df**	**LR stat**	**P**
Intercept (Null model)	-285.15	5			
Season	-272.98	8	3	24.34	<0.001
Season + N	-261.69	9	1	22.6	<0.001
**Season + N + Control**	**-255.44**	**10**	**1**	**12.49**	**<0.001**
c)					
**Tannin Model**	**Log likelihood**	**k**	**LR.df**	**LR stat**	**P**
Intercept (Null model)	-285.15	5			
Season	-272.98	8	3	24.34	<0.001
**Season + Control**	**-267.08**	**9**	**1**	**11.81**	**<0.001**
d)					
**DMD Model**	**Log likelihood**	**k**	**LR.df**	**LR stat**	**P**
Intercept (Null model)	-285.15	5			
Season	-272.98	8	3	24.34	<0.001
**Season + Control**	**-267.08**	**9**	**1**	**11.81**	**<0.001**

Summary of model selection using log ratio statistics, for cumulative link mixed effects modelling of severity of possum browsing on kamahi at line 2. Explanatory variables (season, control, TBA and nutritional variable; a) availN, b) total N, c) tannin effect and d) DMD) were added to the null model of Browse ~ 1 in turn, and the variable with the most explanatory power (determined by log ratio statistic) retained, and the procedure repeated with remaining, unfitted variables. Only the significant model after each iteration is shown (i.e., insignificant models are not shown). The random factors (plot, and tree, nested within plot) were present in all models. The term k refers to the number of coefficients in the model LR.df is the log ratio statistic degrees of freedom LR stat. is the log ratio statistic. P is the P value associated with the log ratio statistic where a P value <0.05 indicates a significant difference between the model and the preceding model (i.e., a significant improvement in the model). The final model is highlighted in bold, defined by the condition that the addition of no other variables or interaction terms produced a significant improvement in model fit.

**Table 4:** Summary of model coefficients, possum browsing on kamahi at Line 2.

**Coefficient**	**Estimate**	**SE**	**Wald Z**	**P**
Summer	-0.693	0.370	-1.87	0.060
Autumn	-0.934	0.386	-2.43	**0.015**
Spring 2011	-1.928	0.407	-4.74	**<0.001**
AvailN	8.067	1.971	4.09	**<0.001**
Control	-2.289	0.567	-4.04	**<0.001**
**Random Factor**	**Variance**			
Plot	0.60			
Plot/Tree	1.06			

Summary of model coefficients from cumulative link mixed effects modelling exploring the fixed effects of season, AvailN and possum control on the severity of browsing by possums on kamahi on Line 2. Note: Spring 2010 and non-control zone are the reference levels. Hence, significant differences (marked in bold text) refer to significant differences from reference levels. Estimate is the coefficient estimate; SE is the standard error of the estimate; Wald Z is the Wald Z statistic; P is the P value associated with the Wald Z statistic. Significant differences from the reference condition are denoted by bold case of the P value.

**Fig 3:**
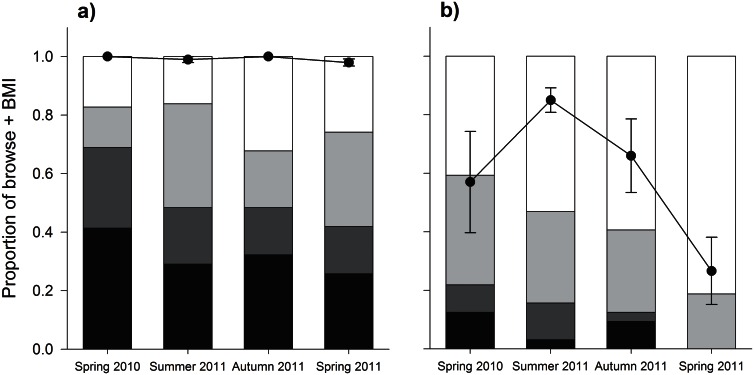
Observations of browsing by possums on kamahi. Proportion of kamahi observations within each browse category for each season in non-control (a) and control (b) zones in the Tararua Mountain Range. The black bar represents the proportion of trees in the heavy browse category, dark grey represents moderate browsing by possums, light grey is light browsing and white represents the proportion of trees with no browsing observed. Also shown, average bite mark index, BMI (filled circles) ± SE (error bars), as a proxy for possum abundance in each season and each control zone. Browse data presented is for 63 kamahi trees at Line 2. BMI data presented is the average BMI per season from ten wax tag transects (20 wax tags per transect) positioned along Line 2.

In the non-control zone of Line 2, browsing and abundance of possums was high over the 12 month period, with bite mark index indicating saturation in all four sampling seasons (Fig 3). However, in the zone where baiting and trapping of possums occurred (control zone), there was a significant decline in the severity and occurrence of browsing by possums on kamahi after control, and the abundance of possums was lower (Fig 3). Over 80% of all trees sampled in the control zone showed no evidence of browsing by possums by Spring 2011, 12 months after possum control began (Fig 3).

**Fig 4:**
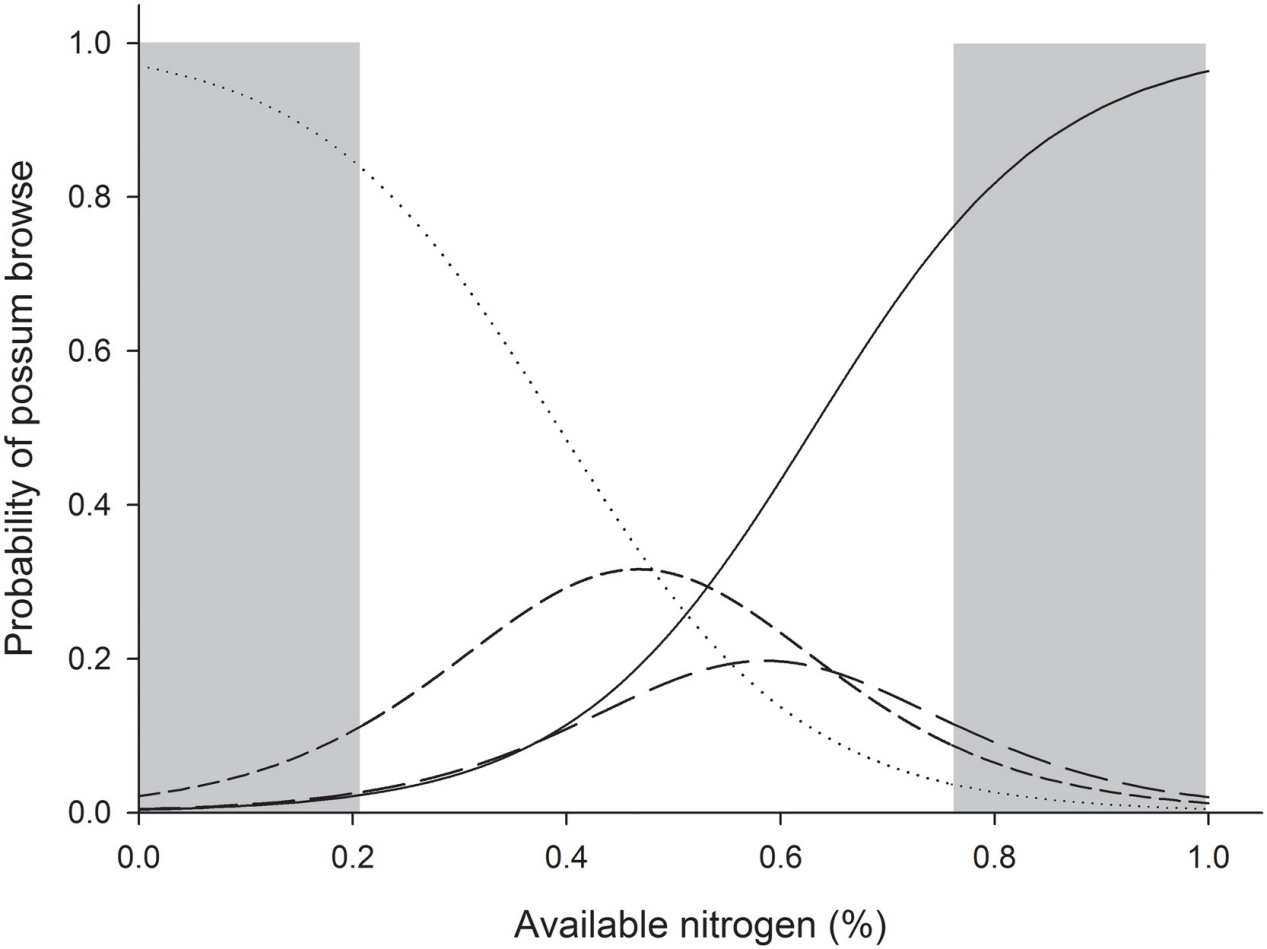
Probability of severity of browsing by possums on kamahi. Modelled probability of severity of browsing by possums on kamahi foliage in response to foliar available nitrogen concentration at Line 2, Tararua Mountain Range. The dotted line represents the probability of no browsing by possums occurring, the short dash is light browse, long dash is moderate browse and solid line is heavy browsing. The figure shows the model for reference levels (Spring 2010, non-control zone; Table 4). Grey boxes denote values beyond the range of available nitrogen observed for kamahi foliage at line 2 in the study (0.20%–0.76%).

Cumulative link mixed effects modeling revealed a strong, positive relationship between kamahi AvailN concentration and extent of browsing by possums (as well as an effect of season and control; Fig 4; Table 4). The likelihood of no browsing occurring decreased as AvailN increased, while light and moderate browsing increased with increasing AvailN, but declined again at high AvailN concentrations. The incidence of heavy damage from browsing by possums was minimal at low AvailN concentrations, but increased rapidly at high AvailN concentrations (Fig 4). Cumulative link mixed model analysis revealed that during Spring 2010 within kamahi at Line 2, even a very small increase in AvailN concentration from 0.3 to 0.5% resulted in an increase in the incidence of heavy possum browse damage from 4% to 17% (Fig 4). At higher AvailN concentrations, the incidence of heavy damage was even greater. For example, there was a 63% chance of observing heavy damage at the maximum measured AvailN concentration of 0.76% and an extrapolated 93% chance of observing heavy browsing damage at 1.0% AvailN concentration, if this was to occur (Fig 4).

## Discussion

We have shown that nutritional quality (AvailN and total N) varies temporally, spatially and among species in a NZ forest and that this variation influences the browsing behaviour of an invasive mammal. Similar sources of variation in foliar nutritional quality have been demonstrated in tropical [39] and temperate forests [40], with implications for populations of native consumers. However, this is the first time that variation in nutritional quality and the corresponding impact of an invasive herbivore has been quantified at a landscape scale. Australian studies have identified nutritional factors, such as total N, the ratio of nitrogen to phenols, plant secondary metabolites (PSMs) such as FPCs as well as AvailN, as important determinants of distribution and local abundance of arboreal marsupials in Australian forests [6–8, 41, 42]. Therefore, the seasonal and spatial distribution of foliar AvailN concentrations in a dominant diet species such as kamahi may be a major determinant of the likely impact of invasive possum populations in NZ, and conceivably in other systems. Thus, this case study provides a foundation for future studies aiming to explain the impacts of invasive herbivorous mammals.

The probability of an individual tree being browsed by possums in the Tararuas increased with foliar AvailN. Specifically, foliar AvailN concentration was related to severity of browsing by possums of kamahi, a tree species considered to be an important food for possums in NZ [15, 26, 32]. Previously, estimates of the within-species tree preferences of possums have been made on the premise that larger trees will be favoured [23,26], but the present study found no relationship between tree basal area and browsing by possums on kamahi. Rather, AvailN was a significant predictor of the browsing decisions of possums. There are many explanations for the relationship between foliar concentrations of AvailN and greater damage from browsing by possums to tree canopies. These explanations include the dietary protein requirements of herbivores for investment in reproduction and lactation as well as in the detoxification of ingested PSMs. Both DeGabriel et al. [29] and McArt et al. [43] found positive relationships between the AvailN concentration of the diet and reproductive success in possums and moose respectively. In addition, several captive studies have shown that protein-rich diets enable animals to eat greater concentrations of PSMs [44–46] although no mechanisms have been proposed. Au et al. [47] further demonstrated the protein costs of detoxification for common brushtail possums. The demonstrated relationship between foliar AvailN and browsing by possums in the present study provides further support for the use of AvailN as an integrative measure that can explain both broad scale and fine scale differences in browsing by possums in NZ within a species where previously, nutritional explanations have only been speculative.

The severity of browsing damage on kamahi varied with season and in response to possum control. The relationship between AvailN concentration and browsing by possums for these trees weakened over the 12 month period following possum control. By Spring 2011, none of the trees in the area where possum abundance had been reduced were observed to be in the ‘moderate’ or ‘heavy’ browse damage categories. Similarly, Duncan et al. [26] found that highly browsed kamahi trees were disproportionately more likely to be found at sites with high possum densities. This, combined with the present study supports the hypothesis that where possum abundance is high, trees with high nutritional quality will suffer the most damage from browsing by possums. However, we note that reduced possum abundance as a result of control is likely to have led to difficulty in assessing browsing damage (ie. a sampling issue). Furthermore, a reduction in the range (and increased mean) of AvailN concentrations for kamahi foliage in Spring 2011 may also explain the weakening in the relationship between AvailN and browsing by possums in this final season.

Seasonal variation in browsing by possums in NZ has previously been attributed to seasonal changes in the relative palatability of foods, complicating the rankings of species according to possum preferences [48, 49]. For example, in the Orongorongo Valley, possums browsed less from rata in Winter and Spring, coinciding with an increase in consumption of supplejack (*Ripogonum scandens*), mahoe and rata vine (*Metrosideros fulgens*) [50]. Thus, a species may be highly damaged by browsing by possums only at certain times of the year. We found seasonal variation in the nutritional quality of foliage, which supports a role for nutrition in these seasonal preferences. Although we found a seasonal effect on browsing by possums (at Line 2), we did not find that possums changed their preferences for tree species (of the five species studied) according to season. Instead, damage to the sampled trees decreased over the 12 month period across all species. Although the sampling design of this experiment may have meant that seasonal changes in browsing of species such as mahoe that were not well represented, or may not have been detectable, we contend that changes in possum abundance (as a result of pest control) had a greater effect on browsing damage by possums than seasonal changes in the nutritional quality of foliage.

We have demonstrated that foliar AvailN can explain the browsing decisions of an invasive mammalian herbivore in a temperate NZ forest. Having demonstrated the link between nutritional quality and severity of browsing damage, these findings can now be incorporated into models aimed at predicting tree mortality due to browsing by possums (e.g.,[21, 23]). Previous studies have demonstrated that nutritional quality at both the home range [29] and landscape-scale [40] regulates herbivore populations. Determining whether populations of invasive herbivores are regulated by similar processes should be a priority.

## Supporting Information

S1 Fig Map of study site in Tararua Mountain Range, New Zealand. Layout of transects (Line 1 –dashed red; and Line 2 –dashed blue) in relation to possum control operations (aerial drop of sodium fluoroacetate baits; within pink boundary) in the Tararua Mountain Range, New Zealand. Copyright in the underlying dataset from which this work has been derived is owned by Greater Wellington Regional Council. Licensed for re-use under the Creative Commons Attribution 3.0 New Zealand license (http://creativecommons.org/licenses/by/3.0/nz/).
(TIF)

S2 Fig Variation in foliar available nitrogen concentration for kamahi. Seasonal variation in the available nitrogen concentration of kamahi foliage at Line 1 (closed circles) and Line 2 (open circles) in the Tararua Mountain Range, New Zealand. Adapted from Windley and Foley (2015).
(TIF)

S1 Table Summary of foliage sampling effort in Tararua Mountain Range. Summary of species sampled in the Tararua Mountain Range New Zealand between Spring 2010 and Spring 2011. Trees (*n*) is the number of trees sampled, and seasons report the number of samples taken from each species.
(DOCX)

S2 Table Significant species-level differences with respect to nutritional quality. Summary of linear mixed effects models on the effect of five New Zealand tree species on 4 nutritional measures (available nitrogen, total nitrogen, tannin effect and dry matter digestibility). In each instance, there were significant differences between species for all four nutritional measures (*n* = 1111).
(DOCX)

S3 Table Summary of main sources of variation in foliar nutritional quality. Final linear mixed effects models identifying the sources of variation in foliar nutrition (AvailN, Total N, Tannin and dry matter digestibility, DMD) in the Tararua Mountain Range, New Zealand. Adapted from Windley and Foley (2015).
(DOCX)

S4 Table Summary of foliar nutritional quality by species and season. Seasonal variation in nutritional quality of five tree species in the Tararua Mountain Range, New Zealand. The same trees were sampled each season. n = number of samples, SD = standard deviation. Nutritional variables are as percentage dry matter.
(DOCX)
