# Supplementary figures and images for: Foliar Nutritional Quality Explains Patchy Browsing Damage Caused by an Invasive Mammal
Source: PLoS One. 2016 May 12;11(5):e0155216. doi: 10.1371/journal.pone.0155216 (PMC4865184; doi:10.1371/journal.pone.0155216)

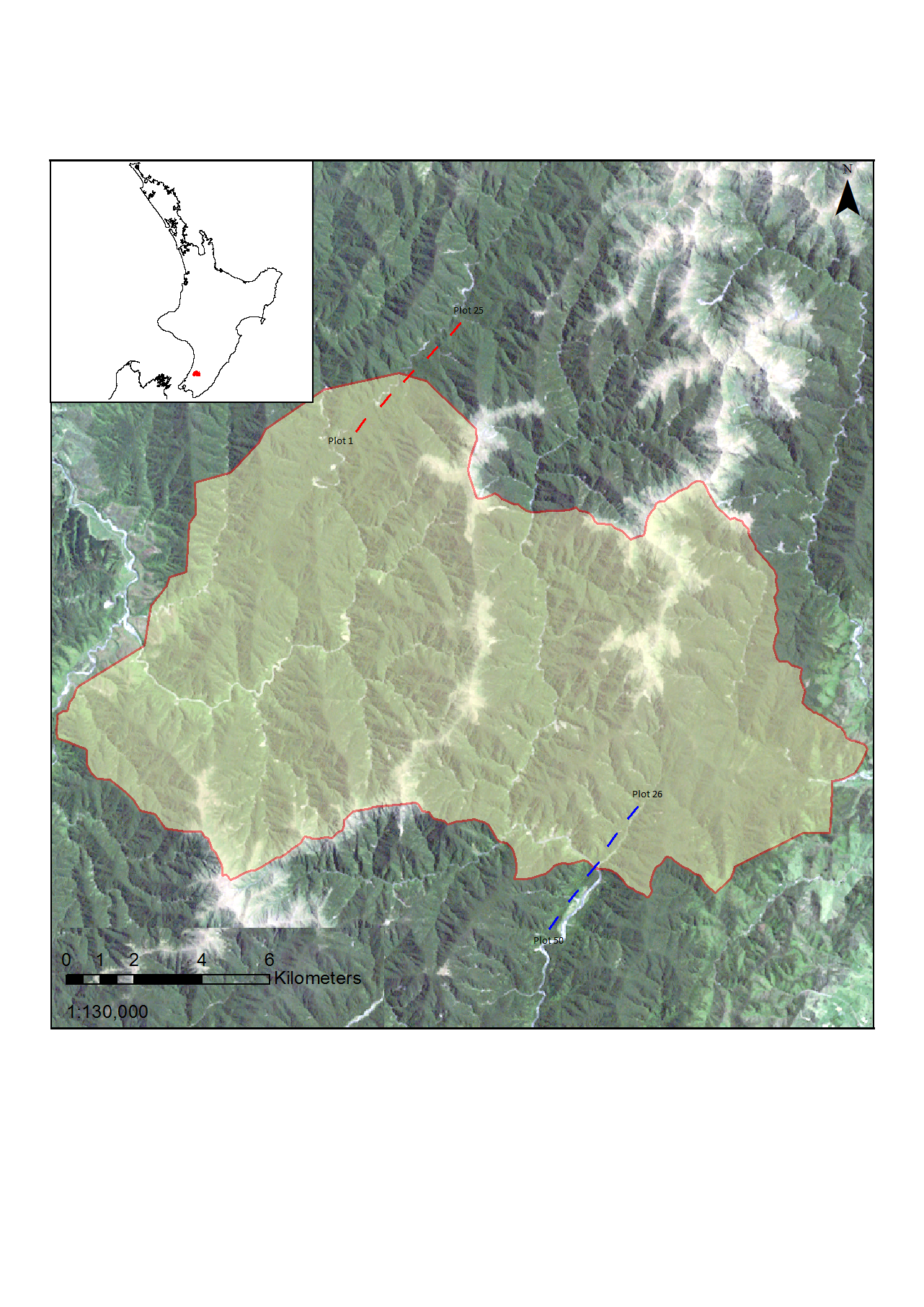

Supplement: S1 Fig — Layout of transects (Line 1 –dashed red; and Line 2 –dashed blue) in relation to possum control operations (aerial drop of sodium fluoroacetate baits; within pink boundary) in the Tararua Mountain Range, New Zealand. Copyright in the underlying dataset from which this work has been derived is owned by Greater Wellington Regional Council. Licensed for re-use under the Creative Commons Attribution 3.0 New Zealand license (http://creativecommons.org/licenses/by/3.0/nz/). (TIF) [file pone.0155216.s001.tif]

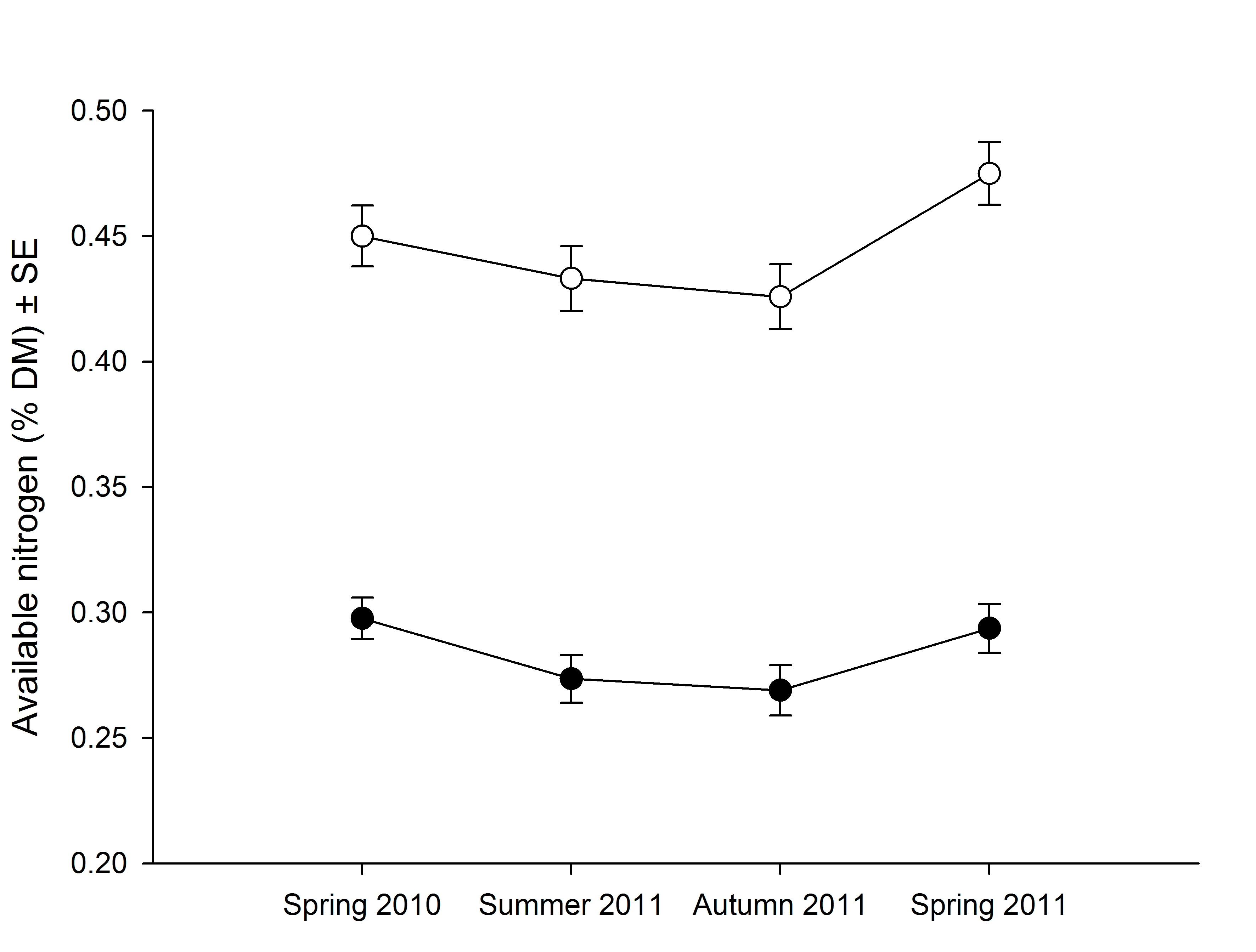

Supplement: S2 Fig — Seasonal variation in the available nitrogen concentration of kamahi foliage at Line 1 (closed circles) and Line 2 (open circles) in the Tararua Mountain Range, New Zealand. Adapted from Windley and Foley (2015). (TIF) [file pone.0155216.s002.TIF]
