# Supplementary material for: Foliar Nutritional Quality Explains Patchy Browsing Damage Caused by an Invasive Mammal
Source: PLoS One. 2016 May 12;11(5):e0155216. doi: 10.1371/journal.pone.0155216 (PMC4865184; doi:10.1371/journal.pone.0155216)
Supplement: S1 Table — Summary of species sampled in the Tararua Mountain Range New Zealand between Spring 2010 and Spring 2011. Trees (n) is the number of trees sampled, and seasons report the number of samples taken from each species. (DOCX) [file pone.0155216.s003.docx]

| **Species** | **Family** | **Common Name** | **Location** | **Trees (*n*)** | Spring 2010 (*n*) | Summer 2011 (*n*) | Autumn 2011 (*n*) | Spring 2011 (*n*) | **Total (*n*)** |
| --- | --- | --- | --- | --- | --- | --- | --- | --- | --- |
| *Weinmannia racemosa* | Cunoniaceae | kamahi | Line 1 & 2 | 128 | 131 | 128 | 128 | 129 | **516** |
| *Myrsine salicina* | Myrsinaceae | toro | Line 1 & 2 | 79 | 85 | 79 | 79 | 79 | **322** |
| *Dacrydium cupressinum* | Podocarpaceae | rimu | Line 1 & 2 | 35 | 40 | 35 | 35 | 35 | **145** |
| *Elaeocarpus dentatus* | Elaeocarpaceae | hinau | Line 2 | 20 | 20 | 20 | 20 | 20 | **80** |
| *Melicytus ramiflorus* | Violaceae | mahoe | Line 2 | 13 | 13 | 13 | 13 | 13 | **52** |
| **Totals** |  |  |  | **275** | **289** | **275** | **275** | **276** | **1115** |
