# Supplementary material for: Foliar Nutritional Quality Explains Patchy Browsing Damage Caused by an Invasive Mammal
Source: PLoS One. 2016 May 12;11(5):e0155216. doi: 10.1371/journal.pone.0155216 (PMC4865184; doi:10.1371/journal.pone.0155216)
Supplement: S2 Table — Summary of linear mixed effects models on the effect of five New Zealand tree species on 4 nutritional measures (available nitrogen, total nitrogen, tannin effect and dry matter digestibility). In each instance, there were significant differences between species for all four nutritional measures (n = 1111). (DOCX) [file pone.0155216.s004.docx]

| **Nutritional parameter** | **Fixed term** | **df** | **SS** | **MS** | **F** | **P** |
| --- | --- | --- | --- | --- | --- | --- |
| Available nitrogen (%) | Species | 5 | 13.216 | 2.6433 | 543.13 | **<0.001** |
| Total nitrogen (%) | Species | 5 | 26.62 | 5.32 | 888.79 | **<0.001** |
| Tannin effect (%) | Species | 5 | 34.64 | 6.93 | 2309.7 | **<0.001** |
| Dry matter digestibility (%) | Species | 5 | 547125 | 109425 | 33849 | **<0.001** |
| Note: Random factor (1\|Plot/Tree) included in each model | | | | | | |
