# Supplementary material for: Foliar Nutritional Quality Explains Patchy Browsing Damage Caused by an Invasive Mammal
Source: PLoS One. 2016 May 12;11(5):e0155216. doi: 10.1371/journal.pone.0155216 (PMC4865184; doi:10.1371/journal.pone.0155216)
Supplement: S3 Table — Final linear mixed effects models identifying the sources of variation in foliar nutrition (AvailN, Total N, Tannin and dry matter digestibility, DMD) in the Tararua Mountain Range, New Zealand. Adapted from Windley and Foley (2015). (DOCX) [file pone.0155216.s005.docx]

| **Nutritional measure** | **Final model** |
| --- | --- |
| AvailN Model | Season+Species+Line+Species*Line+Season*Species+Season* Species*Line |
| Total N Model | Species+Season+Line+Species*Season+Species*Line+Species* Season*Line |
| Tannin Model | Species+Season+Line+Species*Season+Species*Line+Season* Line |
| DMD Model | Species+Season+TBA+Species*Season |
