# Supplementary material for: Foliar Nutritional Quality Explains Patchy Browsing Damage Caused by an Invasive Mammal
Source: PLoS One. 2016 May 12;11(5):e0155216. doi: 10.1371/journal.pone.0155216 (PMC4865184; doi:10.1371/journal.pone.0155216)
Supplement: S4 Table — Seasonal variation in nutritional quality of five tree species in the Tararua Mountain Range, New Zealand. The same trees were sampled each season. n = number of samples, SD = standard deviation. Nutritional variables are as percentage dry matter. (DOCX) [file pone.0155216.s006.docx]

| **Nutritional variable** | **Tree species** | **Spring 2010** | | | | **Summer 2011** | | | | **Autumn 2011** | | | | **Spring 2011** | | | |
| --- | --- | --- | --- | --- | --- | --- | --- | --- | --- | --- | --- | --- | --- | --- | --- | --- | --- |
|  |  | **n** | **Mean** | **SD** | **Range** | **n** | **Mean** | **SD** | **Range** | **n** | **Mean** | **SD** | **Range** | **n** | **Mean** | **SD** | **Range** |
| **Total nitrogen** | KAM | 131 | 0.84 | 0.13 | 0.62–1.23 | 128 | 0.88 | 0.14 | 0.63–1.38 | 128 | 0.92 | 0.14 | 0.68–1.35 | 128 | 0.87 | 0.13 | 0.58–1.21 |
|  | TOR | 85 | 1.06 | 0.15 | 0.78–1.50 | 79 | 1.10 | 0.14 | 0.90–1.50 | 79 | 1.12 | 0.13 | 0.89–1.45 | 79 | 1.05 | 0.16 | 0.79–1.63 |
|  | RIM | 40 | 0.80 | 0.11 | 0.57–1.06 | 35 | 0.81 | 0.11 | 0.61–1.10 | 35 | 0.83 | 0.10 | 0.67–1.02 | 33 | 0.83 | 0.11 | 0.63–1.15 |
|  | HIN | 20 | 1.01 | 0.10 | 0.81–1.23 | 20 | 0.98 | 0.13 | 0.84–1.28 | 20 | 1.03 | 0.14 | 0.84–1.34 | 20 | 1.01 | 0.15 | 0.71–1.34 |
|  | MAH | 13 | 2.03 | 0.25 | 1.67–2.53 | 13 | 2.15 | 0.31 | 1.79–2.73 | 13 | 2.12 | 0.27 | 1.78–2.70 | 13 | 2.11 | 0.35 | 1.63–2.76 |
| **Dry matter digestibility** | KAM | 131 | 56.09 | 1.77 | 50.5–61.4 | 128 | 55.71 | 1.88 | 50.2–59.8 | 128 | 55.47 | 1.83 | 50.3–59.8 | 128 | 57.00 | 1.75 | 53.1–61.5 |
|  | TOR | 85 | 45.95 | 2.50 | 39.3–51.7 | 79 | 44.02 | 2.25 | 38.8–48.9 | 79 | 44.67 | 2.16 | 40.0–49.8 | 79 | 46.65 | 2.41 | 37.2–51.9 |
|  | RIM | 40 | 39.69 | 2.35 | 35.8–45.2 | 35 | 37.02 | 2.86 | 31.4–41.7 | 35 | 36.85 | 2.80 | 31.6–45.3 | 33 | 40.09 | 2.30 | 35.0–45.4 |
|  | HIN | 20 | 47.68 | 1.98 | 44.0–51.3 | 20 | 46.15 | 2.07 | 42.9–49.7 | 20 | 45.11 | 1.63 | 41.4–48.0 | 20 | 47.98 | 2.11 | 44.8–53.4 |
|  | MAH | 13 | 62.28 | 1.84 | 59.8–67.2 | 13 | 61.26 | 1.69 | 58.0–64.5 | 13 | 61.17 | 2.52 | 57.5–65.9 | 13 | 64.59 | 2.61 | 61.2–69.9 |
| **Available nitrogen** | KAM | 131 | 0.37 | 0.11 | 0.11–0.69 | 128 | 0.35 | 0.12 | 0.06–0.76 | 128 | 0.35 | 0.12 | 0.09–0.65 | 128 | 0.38 | 0.12 | 0.06–0.69 |
|  | TOR | 85 | 0.32 | 0.10 | 0.07–0.64 | 79 | 0.26 | 0.11 | 0.06–0.56 | 79 | 0.27 | 0.10 | 0.06–0.55 | 79 | 0.29 | 0.12 | 0.08–0.62 |
|  | RIM | 40 | 0.28 | 0.10 | 0.08–0.58 | 35 | 0.22 | 0.11 | 0.04–0.45 | 35 | 0.23 | 0.09 | 0.04–0.39 | 33 | 0.27 | 0.11 | 0.09–0.67 |
|  | HIN | 20 | 0.66 | 0.09 | 0.47–0.82 | 20 | 0.58 | 0.09 | 0.43–0.74 | 20 | 0.60 | 0.11 | 0.41–0.82 | 20 | 0.63 | 0.12 | 0.39–0.92 |
|  | MAH | 13 | 1.57 | 0.25 | 1.21–2.00 | 13 | 1.62 | 0.31 | 1.21–2.20 | 13 | 1.53 | 0.26 | 1.14–2.03 | 13 | 1.59 | 0.35 | 1.10–2.32 |
| **Available nitrogen +PEG** | KAM | 131 | 0.70 | 0.12 | 0.49–1.07 | 128 | 0.72 | 0.13 | 0.49–1.18 | 128 | 0.74 | 0.13 | 0.51–1.12 | 128 | 0.72 | 0.12 | 0.44–1.01 |
|  | TOR | 85 | 0.82 | 0.13 | 0.59–1.22 | 79 | 0.84 | 0.13 | 0.63–1.21 | 79 | 0.86 | 0.11 | 0.62–1.15 | 79 | 0.80 | 0.14 | 0.58–1.31 |
|  | RIM | 40 | 0.48 | 0.11 | 0.25–0.78 | 35 | 0.46 | 0.12 | 0.23–0.78 | 35 | 0.48 | 0.10 | 0.32–0.66 | 33 | 0.50 | 0.11 | 0.28–0.85 |
|  | HIN | 20 | 0.75 | 0.10 | 0.57–0.96 | 20 | 0.71 | 0.11 | 0.56–0.96 | 20 | 0.75 | 0.13 | 0.58–1.05 | 20 | 0.74 | 0.14 | 0.46–1.06 |
|  | MAH | 13 | 1.77 | 0.25 | 1.41–2.25 | 13 | 1.87 | 0.30 | 1.50–2.43 | 13 | 1.84 | 0.26 | 1.50–2.38 | 13 | 1.83 | 0.35 | 1.37–2.51 |
